# Choline fenofibrate add-on to statins in persistent hypertriglyceridemia: efficacy, apoB-containing lipoprotein remodeling, and renal function

**DOI:** 10.1016/j.athplu.2026.100622

**Published:** 2026-10-30

**Authors:** Byung Jin Kim, Hyeon Ju Jeong, Eun Seok Kang, Min-Woong Kim, Sang-Hyun Kim, Yong-Hyun Kim, Ki Hong Lee, Geu-Ru Hong, Young Sup Byun, Hoyoun Won, Dong Heon Yang, Jae Hyuk Lee, Myung Soo Park, Se Eun Park, Sung Uk Kwon, Si-Hyuck Kang, Min Seop Song

**Affiliations:** a Division of Cardiology, Department of Internal Medicine, Kangbuk Samsung Hospital, Sungkyunkwan University School of Medicine, Seoul, South Korea; b Division of Cardiology, Department of Internal Medicine, Myongji Hospital, Hanyang University Medical Center, Goyang, South Korea; c Department of Internal Medicine, Yonsei University College of Medicine, Seoul, South Korea; d Department of Cardiology, Changwon Hanmaeum Hospital, Hanyang University College of Medicine, Changwon, South Korea; e Cardiology, Internal Medicine, Seoul Boramae Hospital, Seoul National University College of Medicine, Seoul, South Korea; f Endocrinology, Bundang Jesaeng Hospital, Seongnam, South Korea; g Chonnam National University Medical School & Hospital, Gwangju, South Korea; h Division of Cardiology, Severance Cardiovascular Hospital, Yonsei University College of Medicine, Seoul, South Korea; i Division of Cardiology, Department of Internal Medicine, Sanggye Paik Hospital, Inje University College of Medicine, Seoul, South Korea; j Cardiovascular & Arrhythmia Center, Chung-Ang University Hospital, Chung-Ang University College of Medicine, Seoul, South Korea; k Division of Cardiology, Department of Internal Medicine, Kyungpook National University Hospital, Daegu, South Korea; l Division of Endocrinology and Metabolism, Department of Internal Medicine, Myongji Hospital, Goyang, South Korea; m Division of Cardiology, Department of Internal Medicine, Dongtan Sacred Heart Hospital, Hwaseong, South Korea; n Division of Endocrinology and Metabolism, Department of Internal Medicine, Kangbuk Samsung Hospital, Sungkyunkwan University School of Medicine, Seoul, South Korea; o Department of Internal Medicine, Ilsan Paik Hospital, Inje University College of Medicine, Goyang, South Korea; p Department of Internal Medicine and Cardiovascular Center, Seoul National University Bundang Hospital, Seoul, South Korea; q Dr Song's Clinic, Seoul, South Korea

**Keywords:** Apolipoprotein B, Cystatin C, Fenofibrate, Remnant cholesterol, Renal safety, Small dense LDL, Triglyceride

## Abstract

**Background:**

Fenofibrate added to statin therapy lowers triglycerides (TG), but its effects on apolipoprotein B (apoB)-containing lipoproteins and renal function remain incompletely characterized.

**Objective:**

To evaluate lipid and lipoprotein changes and renal function during choline fenofibrate add-on therapy in statin-treated patients with persistent hypertriglyceridemia.

**Methods:**

In this 26-center prospective observational study, adults with TG ≥ 200 mg/dL on statin therapy were evaluated at baseline, Week 12, and Week 24. Lipoprint analysis and cystatin C were available in subsets.

**Results:**

Among 882 patients, TG decreased by 48.9% at Week 24 (p < 0.001), whereas low-density lipoprotein cholesterol (LDL-C) increased by 7.1 mg/dL. ApoB did not change significantly in participants with paired measurements (n = 132). In the paired Lipoprint subset (n = 62), small-dense LDL (sdLDL) fraction decreased from 6.84% to 3.80% and mean LDL particle size increased from 259.3 to 263.4 Å (both p < 0.001). Creatinine-based estimated glomerular filtration rate (eGFR) declined during treatment; cystatin C-based eGFR showed a similar direction in a smaller paired subset (n = 135).

**Conclusions:**

Choline fenofibrate add-on therapy markedly reduced TG and was accompanied by lower sdLDL fraction and larger LDL particle size despite a modest rise in LDL-C. ApoB and cystatin C findings were supportive but limited to subsets. Renal function should be monitored during treatment.

## Highlights

• Choline fenofibrate add-on to statin lowered triglycerides and remnant cholesterol in persistent hypertriglyceridemia.
• Paired Lipoprint data showed lower sdLDL fraction and larger LDL particle size at Week 24.
• LDL-C rose modestly; apoB did not change significantly in the smaller paired subset.
• Creatinine-based eGFR declined during treatment; cystatin C findings were directionally similar in a smaller subset.

## Graphical abstract

**Figure:**
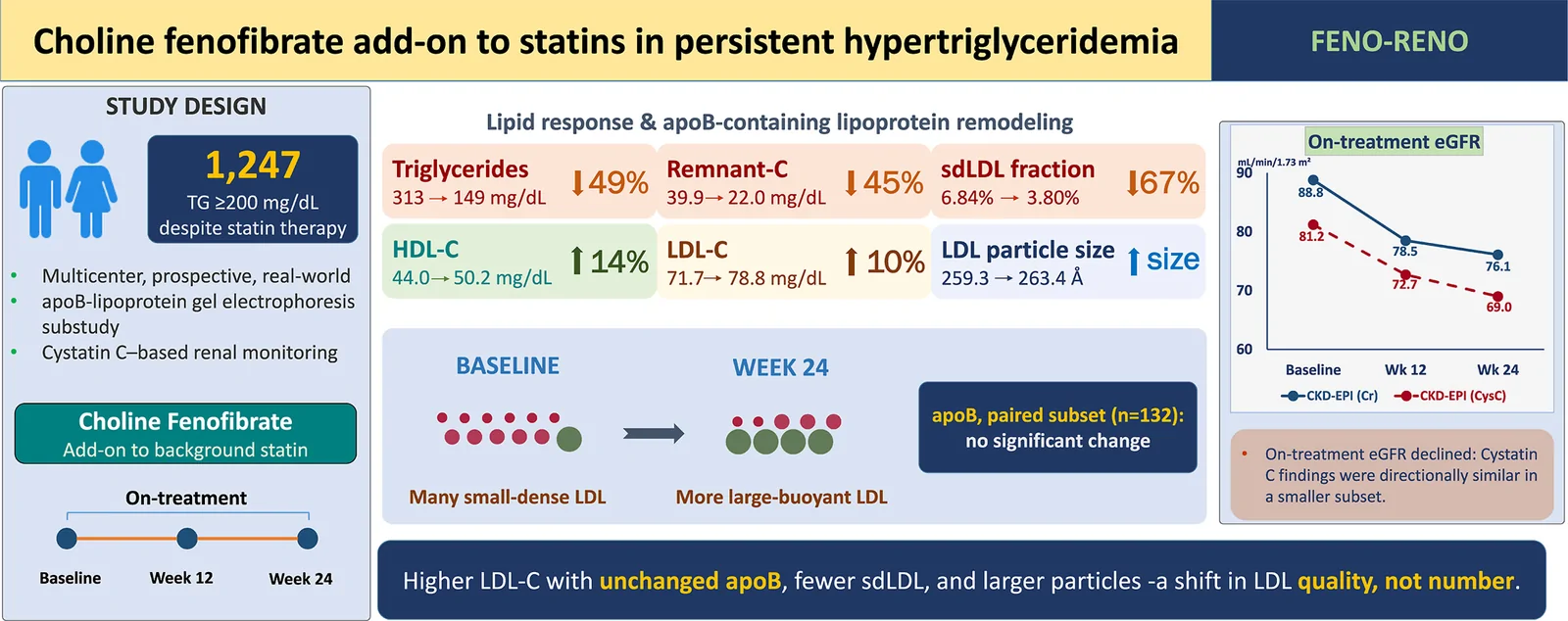

## 1 Introduction

Statin therapy has markedly improved cardiovascular outcomes through effective low-density lipoprotein-cholesterol (LDL-C) lowering, yet substantial residual risk remains even among patients who achieve recommended LDL-C targets [1]. Part of this residual risk is attributed to triglyceride (TG)-rich lipoproteins and their remnants, which carry cholesterol, penetrate the arterial wall [2], and have been causally linked to atherosclerosis by human genetic studies [3], [4]. Elevated TG is therefore recognized as a marker of increased cardiovascular risk, and persistent hypertriglyceridemia despite statin therapy remains a common clinical challenge [2], [5].

Despite this mechanistic rationale, the clinical evidence for fibrate therapy has been inconsistent. Earlier trials suggested cardiovascular benefit, particularly in patients with high TG and low high-density lipoprotein cholesterol (HDL-C) [6], [7], whereas more recent studies conducted in the era of background statin therapy have shown neutral findings [8], [9], [10]. International guidelines reflect this uncertainty and differ in the extent to which fibrates are recommended for patients with persistent hypertriglyceridemia on statin therapy [11], [12], [13], [14], [15]. Nevertheless, fenofibrate continues to be used in routine practice for patients with mixed dyslipidemia or isolated hypertriglyceridemia, especially when additional risk reduction beyond LDL-C lowering is considered necessary.

Renal safety is another issue in fenofibrate therapy. Serum creatinine commonly rises during treatment and declines after discontinuation, a pattern generally regarded as functional and reversible [16], [17], [18]. However, creatinine is influenced by muscle mass, dietary intake, and tubular secretion, and changes in creatinine alone do not necessarily indicate whether glomerular filtration rate (GFR) has truly changed [17], [19]. Cystatin C is less affected by non-renal factors and provides a complementary estimate of GFR, but data on cystatin C during fenofibrate therapy remain limited [20], [21], [22].

Choline fenofibrate is a fenofibrate formulation designed to improve hydrophilicity and bioavailability, with the practical advantage of administration independent of meals [23]. Previous studies have suggested that adding choline fenofibrate to statin therapy improves TG and HDL-C levels in patients whose TG concentrations remain elevated despite statin treatment [24], [25]. However, data on its longer-term effectiveness and renal safety in routine clinical practice are still limited. We therefore conducted a multicenter, prospective observational study to evaluate lipid and lipoprotein changes and renal function during choline fenofibrate add-on therapy in patients with persistent hypertriglyceridemia under real-world conditions. ApoB, Lipoprint, and cystatin C measurements were analyzed when available.

## 2 Methods

### **2.1** *Study Design and population*

This multicenter, single-arm, prospective, observational study enrolled adults with dyslipidemia and inadequately controlled hypertriglyceridemia at 26 centers in Korea between June 15, 2022 and May 21, 2025. Clinical assessments, treatment decisions, laboratory testing, and follow-up were performed during routine outpatient care at the discretion of the treating investigator. Routine biochemical measurements were performed at the local laboratories of the participating centers; no central reference laboratory was used. The protocol did not mandate a common analytical platform or reagent across centers, and site-specific assay methods were not collected. Accordingly, inter-laboratory variability could not be quantified. Concomitant medications were recorded during follow-up, but clinically necessary treatment changes were not prohibited. Lifestyle counseling for hypertriglyceridemia, including dietary modification, weight management, physical activity, and moderation of alcohol intake, was provided as part of usual care according to the treating physician's judgment and was not standardized by protocol. The protocol was approved by the institutional review board at each participating center (IRB No. 2022-03-051, Kangbuk Samsung Hospital). All participants provided written informed consent in accordance with the Declaration of Helsinki and International Council for Harmonization Good Clinical Practice guidelines.

Eligible participants were ≥ 19 years of age, had been receiving statin therapy for ≥ 4 weeks, had a baseline TG ≥ 200 mg/dL, and were judged by the investigator to be appropriate candidates for additional choline fenofibrate therapy. Key exclusion criteria were fibrate or omega-3 fatty acid use within 2 months before baseline; hypersensitivity to choline fenofibrate; aspartate aminotransferase or alanine aminotransferase ≥ 3× the upper limit of normal; estimated glomerular filtration rate (eGFR) < 30 mL/min/1.73 m^2^; a current or prior history of myopathy including rhabdomyolysis; or any other condition considered unsuitable for study participation. Choline fenofibrate was prescribed at the labeled dose (135 mg fenofibric acid equivalent once daily), independent of meals.

### 2.2 Endpoints

The primary endpoint was the percent change in TG from baseline to Week 24. Secondary endpoints included the percent change in TG at Week 12; changes in total cholesterol (TC), non-HDL-C, HDL-C, LDL-C, remnant cholesterol, apolipoprotein A1 (apoA1), apoB, lipoprotein(a), and derived lipid ratios (TC/HDL-C, LDL-C/HDL-C, non-HDL-C/HDL-C, TG/HDL-C, apoB/apoA1, apoB/LDL-C) at Weeks 12 and 24; and risk-stratified attainment of the prespecified non-HDL-C and LDL-C targets (Supplementary Fig. 1). Non-HDL-C was calculated as TC minus HDL-C, and remnant cholesterol as TC minus HDL-C minus LDL-C. Goal-attainment analyses were prespecified for LDL-C and non-HDL-C only; no target-attainment endpoints were defined for the other lipid parameters.

An exploratory substudy characterized LDL particle size and the sdLDL fraction using polyacrylamide gel electrophoresis (Lipoprint LDL system, Quantimetrix Corp, Redondo Beach, CA, USA) at baseline and Week 24 in participants for whom the additional assessment was available. Analyses of change were restricted to participants with measurements at both time points. Insulin resistance was assessed by homeostatic model assessment of insulin resistance (HOMA-IR), homeostatic model assessment of beta-cell function (HOMA-β), and quantitative insulin sensitivity check index (QUICKI). HOMA-IR was calculated as fasting insulin (μU/mL) × fasting glucose (mg/dL)/405; HOMA-β as 360 × fasting insulin (μU/mL)/[fasting glucose (mg/dL) − 63]; and QUICKI as 1/[log(fasting insulin) + log(fasting glucose)]. The prevalence of metabolic syndrome was tracked at each visit.

Safety endpoints included adverse events, adverse drug reactions (ADRs), serious adverse events (SAEs), prespecified adverse events of special interest (major adverse cardiovascular events and COVID-19 infection), and renal function. Renal function was characterized by serum creatinine, cystatin C, and eGFR derived from the chronic kidney disease epidemiology collaboration (CKD-EPI) creatinine, CKD-EPI cystatin C, and CKD-EPI creatinine–cystatin C equations. A prespecified renal safety follow-up at Week 36 (±4 weeks) was offered to participants with a serum creatinine increase ≥ 1.5× baseline or an eGFR decrease ≥ 25% at Week 24; this visit was performed approximately 12 weeks after choline fenofibrate discontinuation.

### 2.3 Statistical analysis

The safety set comprised all participants who received at least one dose of study drug. The full analysis set (FAS) comprised safety-set participants with at least one post-baseline efficacy assessment. The per-protocol set (PPS) comprised FAS participants who completed 24 weeks of treatment without major protocol deviations. The primary efficacy analysis was performed in the FAS. Continuous variables are summarized as mean (SD) or median [IQR] as appropriate. Within-participant changes were assessed using the paired *t*-test or Wilcoxon signed-rank test, as appropriate. Categorical variables were compared using McNemar's test. No imputation was applied for missing data, and all analyses were based on observed data. For risk-stratified lipid goal attainment, the denominator at each visit comprised participants with an evaluable cardiovascular-risk category and an evaluable value for the relevant lipid parameter at that visit; missing values were excluded from the denominator and were not classified as treatment failures. Two-sided p-values <0.05 were considered statistically significant, and analyses were performed with SAS version 9.4 (SAS Institute, Cary, NC, USA). As a post hoc renal sensitivity analysis, hypertension was defined as baseline systolic blood pressure ≥140 mmHg, diastolic blood pressure ≥90 mmHg, or use of antihypertensive medication; diabetes mellitus was defined as fasting plasma glucose ≥126 mg/dL, HbA1c ≥ 6.5%, or use of antidiabetic medication. Change in creatinine-based eGFR from baseline to Week 24 was compared between participants with and without each condition using Welch's *t*-test. A multivariable linear regression model of eGFR change included baseline eGFR, age, sex, hypertension, and diabetes mellitus. Classification was based on available baseline data; participants whose status could not be determined were excluded from the corresponding analysis. As an additional post hoc sensitivity analysis, concomitant lipid-modifying medication records from baseline through the Week 24 assessment were reviewed. The frequency and type of background lipid-modifying therapy changes were summarized, and the principal lipid and creatinine-based renal outcomes were reanalyzed after excluding participants with any recorded change in background lipid-modifying therapy before the Week 24 assessment. No imputation was applied.

## 3 Results

### 3.1 Disposition and baseline characteristics

Of 1247 enrolled participants, 1224 (98.2%) received at least one dose and formed the safety set, 882 (72.1% of the safety set) were included in the FAS, and 575 (65.2% of the FAS) comprised the PPS (Supplementary Fig. 2). Baseline characteristics of the FAS are shown in Table 1. The mean age was 61.5 years and 65.9% were male. Among 877 participants with an evaluable risk category, 70.2% were classified as high or very-high cardiovascular risk according to the 2018 Korean dyslipidemia guidelines. Metabolic syndrome was present in 750 of 877 evaluable participants (85.5%) at baseline. All participants were receiving statin therapy by study eligibility.

**Table 1:** Baseline characteristics of the full analysis set (n = 882).

Variables	Values
Age, mean(SD), year	61.5 (12.2)
Male, N (%)	581/882 (65.9)
Body mass index, mean (SD), kg/m^2^	26.7 (3.9)
Waist circumference, mean (SD), cm	90.6 (10.0)
Smoking, n (%)	
Never	491/863 (56.9)
Former	167/863 (19.4)
Current	205/863 (23.8)
Alcohol Drinking, n(%)	
Never	417/862 (48.4)
Former	67/862 (7.8)
Current	378/862 (43.9)
Duration of Dyslipidemia, mean (SD), months	68.0 (67.1)
Cardiovascular risk category^a^, n (%)	
Low	62/877 (7.1)
Moderate	200/877 (22.8)
High	254/877 (29.0)
Very high	361/877 (41.2)
Number of major risk factors, mean (SD)	2.3 (0.9)
Comorbidity, N (%)	
Hypertension	586/882 (66.4)
Diabetes mellitus	283/882 (32.1)
Angina pectoris	151/882 (17.1)
Myocardial infarction	50/882 (5.7)
Peripheral artery disease	16/882 (1.8)
Cerebral infarction	26/882 (2.9)
Metabolic Syndrome, N (%)	750/877 (85.5)
Waist circumference (men ≥90 cm, women ≥85 cm)	224/877 (25.5)
Triglycerides ≥150 mg/dL	877/877 (100.0)
Blood pressure ≥130/85 mmHg or antihypertensive medication	785/877 (89.5)
Fasting glucose ≥100 mg/dL or hypoglycemic medication	629/877 (71.7)
HDL-C (men <40 mg/dL, women <50 mg/dL)	429/877 (48.9)
Number of metabolic syndrome component, mean (SD)	3.36 (0.85)

Data are presented as mean (standard deviation) or n/N (percentage) for categorical variables. N denotes the number of participants with evaluable data for the corresponding variable. Background lipid-lowering regimen rows from the enrolled population were removed so that Table 1 consistently represents the FAS.

HDL, high-density lipoprotein; SD, standard deviation.

a Per 2018 Korean guidelines for the management of dyslipidemia.^15^ Low-risk group was defined as individuals with no major risk factor; intermediate-risk group was defined as individuals with one or more major risk factors. High HDL-cholesterol (≥60 mg/dL) is considered a protective factor, so one risk factor is deducted.

### 3.2 Lipid response and remodeling of apoB-containing lipoproteins

In the FAS, mean TG decreased from 313.4 (158.2) mg/dL at baseline to 161.8 (181.2) mg/dL at Week 12 and 148.7 (157.0) mg/dL at Week 24, corresponding to mean percent changes of −46.3% and −48.9%, respectively (both p < 0.001). The reduction was evident by Week 12 and sustained through Week 24, and was consistent in the PPS (mean TG 310.8 to 141.7 mg/dL at Week 24; mean percent change −50.3%, p < 0.001). Remnant cholesterol decreased from 39.9 to 22.0 mg/dL at Week 24 (p < 0.001), non-HDL-C from 114.3 to 101.3 mg/dL (p < 0.001), and HDL-C rose from 44.0 to 50.2 mg/dL (p < 0.001; Table 2, Fig. 1).

**Table 2:** Changes in mean lipid parameters from baseline to Week 12 and 24.

Variables	Mean Lipid levels, mg/dL		
	Baseline	Week 12	Week 24
Triglycerides, mg/dL	313.37 (158.18)	161.84 (181.17)^a^	148.66 (157.00)^b^
Remnant cholesterol, mg/dL	39.92 (19.03)	22.55 (15.95)^a^	22.03 (11.53)^b^
Total cholesterol, mg/dL	158.18 (31.95)	149.61 (28.94)^a^	151.47 (29.35)^b^
Non-HDL-C, mg/dL	114.25 (30.09)	99.98 (28.50)^a^	101.29 (28.77)^b^
HDL-C, mg/dL	43.98 (10.10)	49.70 (12.36)^a^	50.18 (12.43)^b^
LDL-C, mg/dL	71.73 (27.04)	76.85 (23.92)^a^	78.81 (24.96)^b^
Apolipoprotein A1, mg/dL	145.73 (22.17)	NA	149.43 (29.34)
Apolipoprotein B, mg/dL	79.57 (18.89)	NA	79.31 (18.65)
Lipoprotein(a), nmol/L	34.78 (56.13)	NA	35.11 (46.89)

Values are presented as mean (SD). Evaluable n at baseline/Week 12/Week 24 was 882/559/765 for triglycerides; 726/487/641 for remnant cholesterol; 838/541/742 for total cholesterol; 842/543/734 for non-HDL-C; 871/555/756 for HDL-C; 861/550/761 for LDL-C; 151/NA/158 for apoA1; 168/NA/168 for apoB; and 162/NA/160 for lipoprotein(a). For apoB, paired baseline and Week 24 measurements were available in 132 participants. Statistical significance was assessed using paired *t*-test or Wilcoxon signed-rank test, as appropriate. Implausible negative calculated LDL-C or remnant-cholesterol values were excluded from summaries of those variables.

HDL-C, high-density lipoprotein-cholesterol; LDL-C, low-density lipoprotein-cholesterol; NA, non-applicable.

a Statistically significant difference between baseline and Week 12 (p < 0.05).

b Statistically significant difference between baseline and Week 24 (p < 0.05).

**Fig. 1:**
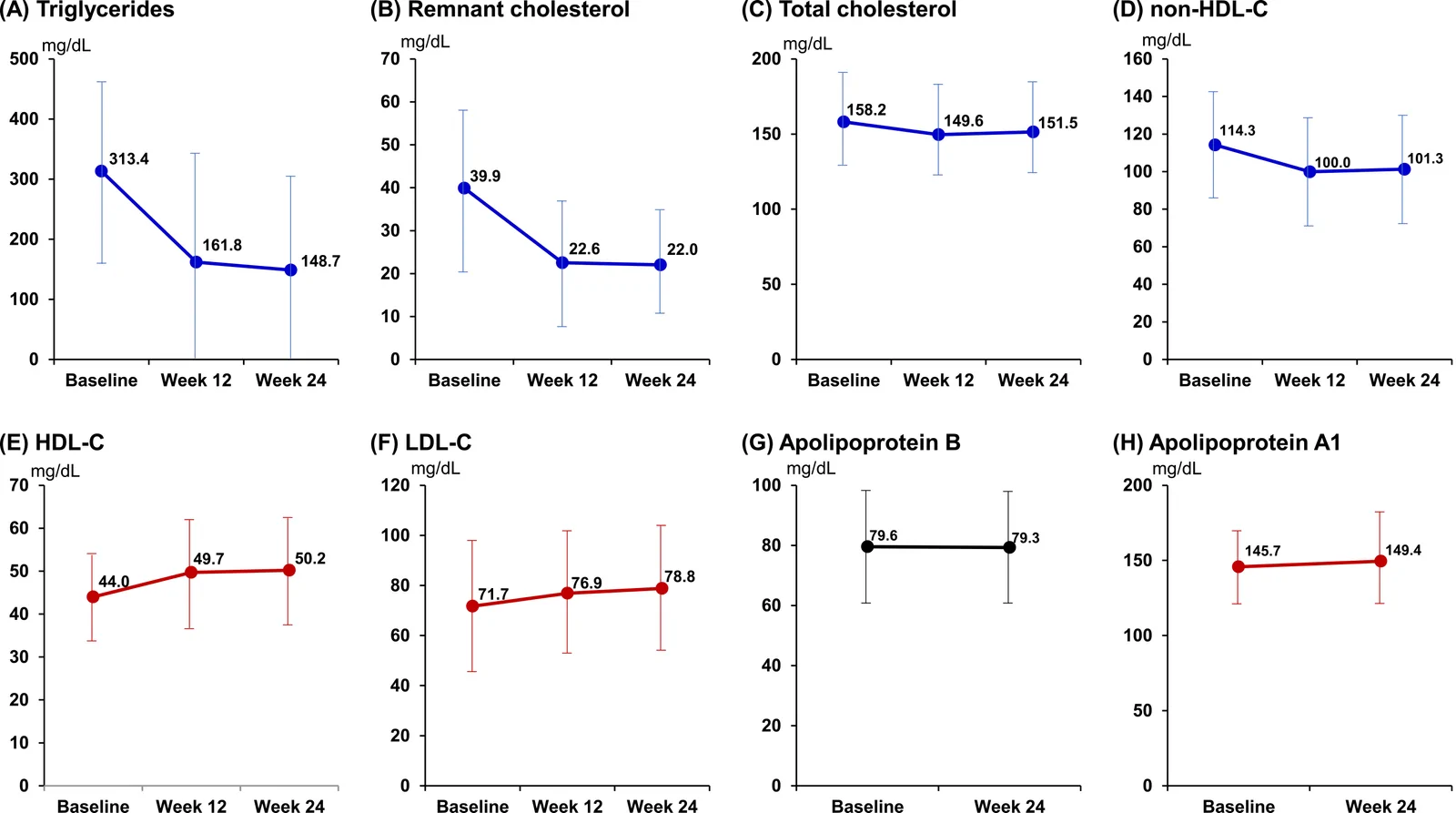
Serum concentrations of lipid parameters at baseline, Week 12, and Week 24 Values are mean (SD); error bars indicate SD. The y-axes begin at zero to avoid visually exaggerating the magnitude of change. Panels G and H show baseline and Week 24 data only. Evaluable sample sizes for each lipid and visit are provided in Table 2. HDL-C, high-density lipoprotein-cholesterol; LDL-C, low-density lipoprotein-cholesterol.

LDL-C increased modestly from 71.7 to 78.8 mg/dL at Week 24 (+7.1 mg/dL; p < 0.001) (Table 2). ApoB measurements at both baseline and Week 24 were available in 132 participants; in this paired subset, apoB did not change significantly (p = 0.977). ApoA1 and lipoprotein(a) were also available in smaller subsets (Table 2). Lipid ratios are presented in Supplementary Table 1.

In the exploratory Lipoprint substudy, 62 participants had measurements at both baseline and Week 24. Mean sdLDL fraction decreased from 6.84 (4.58)% to 3.80 (4.90)% (mean absolute change, −3.04 [4.16] percentage points; paired *t*-test p < 0.001). Among the 58 participants with baseline sdLDL >0, the median individual relative change was −66.9%. Mean LDL particle size increased from 259.3 (10.4) to 263.4 (14.9) Å (mean change, +4.11 Å; Wilcoxon signed-rank p < 0.001; Fig. 2). An illustrative paired profile from the same participant is shown in Supplementary Fig. 3.

**Fig. 2:**
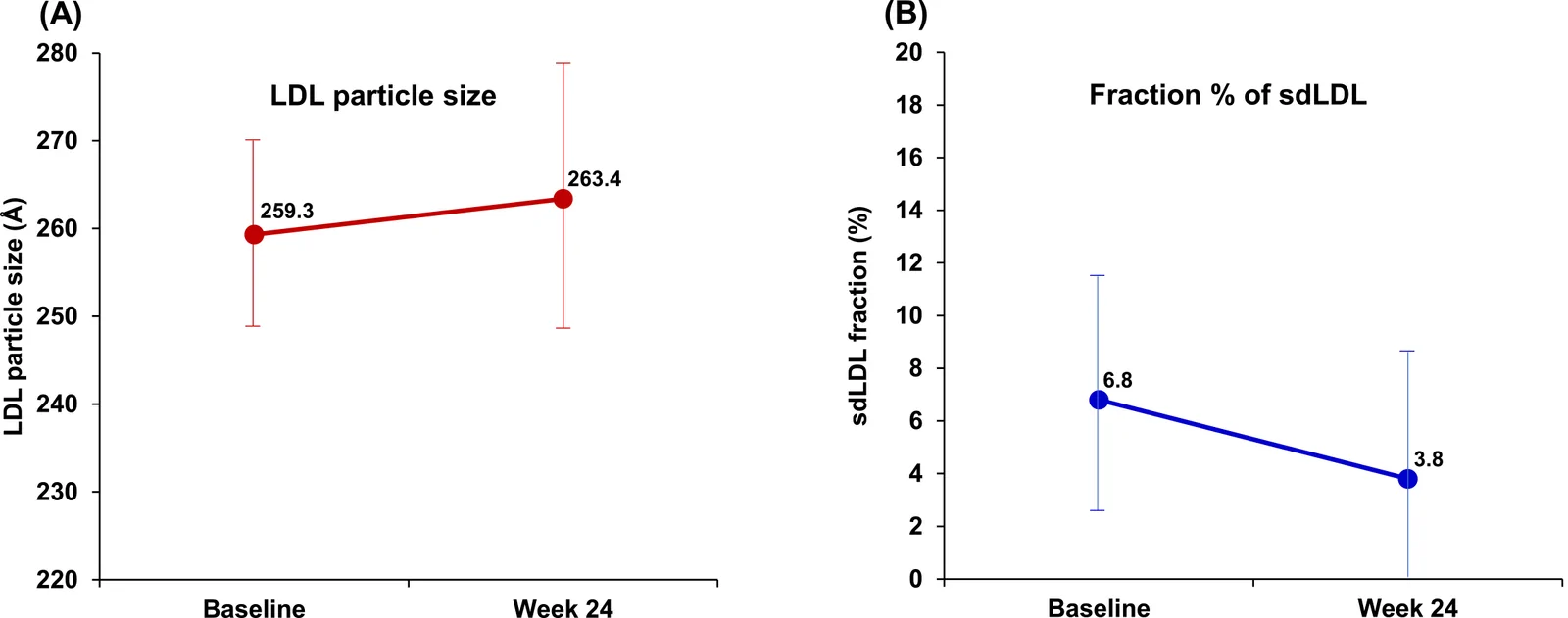
Paired changes from baseline to Week 24 in LDL particle size and sdLDL fraction in the exploratory Lipoprint substudy (n = 62) Values are mean (SD); error bars indicate SD. Mean LDL particle size increased from 259.3 (10.4) Å to 263.4 (14.9) Å, whereas mean sdLDL fraction decreased from 6.84 (4.58)% to 3.80 (4.90)%. Both changes were significant (LDL particle size, Wilcoxon signed-rank p < 0.001; sdLDL fraction, paired *t*-test p < 0.001). LDL, low-density lipoprotein; sdLDL, small dense LDL.

Among 847 FAS participants who attended the Week 24 visit, concomitant lipid-modifying medication data were evaluable in 841 (99.3%). Fifty-four participants (6.4%) had at least one recorded change before the Week 24 assessment, whereas 787 (93.6%) had no recorded change. Fifty-one participants had changes involving statin and/or ezetimibe therapy. In the 787 participants with stable background lipid-modifying therapy, the major lipid findings were consistent with the primary analysis: TG decreased by 163.8 mg/dL (paired n = 712; p < 0.001), LDL-C increased by 7.6 mg/dL (n = 697; p < 0.001), and apoB changed by +0.8 mg/dL (n = 120; p = 0.959) (Supplementary Table 6).

### 3.3 Risk-stratified lipid goal attainment

Using visit-specific evaluable denominators, non-HDL-C target attainment at baseline, Week 12, and Week 24 was 45.0% (157/349), 63.1% (154/244), and 58.2% (177/304) in the very-high-risk group; 75.7% (187/247), 85.1% (137/161), and 85.9% (189/220) in the high-risk group; 90.4% (170/188), 96.3% (104/108), and 95.0% (153/161) in the moderate-risk group; and 98.3% (57/58), 96.7% (29/30), and 100.0% (49/49) in the low-risk group. Corresponding LDL-C target attainment was 61.0% (216/354), 52.0% (127/244), and 45.5% (141/310); 85.0% (209/246), 83.2% (134/161), and 84.6% (192/227); 95.4% (188/197), 97.2% (106/109), and 94.1% (159/169); and 100.0% (62/62), 96.9% (31/32), and 98.1% (51/52), respectively (Supplementary Fig. 4).

### 3.4 Metabolic syndrome and insulin-resistance indices

Among 840 FAS participants with metabolic syndrome status evaluable at both visits, the prevalence fell from 85.1% at baseline to 39.5% at Week 24 (p < 0.001); 395 of 715 participants who had metabolic syndrome at baseline no longer met the criteria at Week 24 (47.0% resolution; Supplementary Fig. 5). Fasting plasma glucose decreased (124.3 to 117.3 mg/dL, p < 0.001), and HbA1c also decreased slightly but significantly from 6.76% to 6.61% at Week 24 (p < 0.001). In participants with paired data, HOMA-IR improved from 3.53 to 2.80 (p = 0.015) and QUICKI increased from 0.32 to 0.33 (p = 0.009), whereas HOMA-β did not change significantly (p = 0.433) (Supplementary Table 2).

### 3.5 Safety

In the safety set (n = 1224), any adverse event was reported in 90 participants (7.35%), adverse drug reactions in 19 (1.55%), and serious adverse events in 9 (0.74%); there were no serious adverse drug reactions and no deaths (Supplementary Table 3). Adverse events of special interest were rare. Among selected chemistry measures, ALT and ALP decreased during treatment, uric acid decreased from 5.64 (1.48) to 4.49 (1.36) mg/dL at Week 24 (p < 0.001), and CK increased modestly from 117.57 (74.92) to 132.22 (104.34) U/L (p = 0.004) (Supplementary Table 4). Other changes were small or inconsistent.

### 3.6 Renal safety: on-treatment changes

In the safety set, creatinine-based eGFR decreased from 88.8 to 78.5 mL/min/1.73 m^2^ at Week 12 and to 76.1 mL/min/1.73 m^2^ at Week 24 (both p < 0.001; Table 3). Cystatin C-based estimates were available in substantially smaller subsets. Among 135 participants with paired baseline and Week 24 cystatin C-based eGFR, the mean value decreased from 80.4 to 69.2 mL/min/1.73 m^2^ (p < 0.001), with a similar direction for the combined creatinine-cystatin C equation. In the post hoc analysis of creatinine-based eGFR, the mean Week 24 change was −12.10 (9.96) mL/min/1.73 m^2^ in participants with hypertension (n = 486) and −12.02 (10.25) mL/min/1.73 m^2^ in those without hypertension (n = 186; p = 0.930). The corresponding changes were −11.76 (10.21) and −12.20 (9.88) mL/min/1.73 m^2^ in participants with and without diabetes mellitus, respectively (p = 0.582). In the multivariable model (n = 638), neither hypertension nor diabetes mellitus was associated with Week 24 eGFR change (Supplementary Table 5). In the stable background lipid-therapy sensitivity population, creatinine-based eGFR decreased by 12.1 mL/min/1.73 m^2^ (paired n = 633; p < 0.001), consistent with the primary analysis (Supplementary Table 6).

**Table 3:** Changes in renal function from baseline to Week 12 and Week 24 in the safety set.

Parameter	Baseline	Week 12	p-value^a^	Week 24	p-value^b^
eGFR (CKD-EPI creatinine), mL/min/1.73 m^2^	88.79 (17.26)	78.51 (20.26)	< 0.001	76.06 (20.18)	< 0.001
eGFR (CKD-EPI cystatin C), mL/min/1.73 m^2^	81.15 (24.49)	72.68 (22.80)	< 0.001	69.00 (21.60)	< 0.001
eGFR (CKD-EPI creatinine–cystatin C), mL/min/1.73 m^2^	87.48 (22.34)	79.51 (22.13)	< 0.001	74.78 (20.72)	< 0.001
Serum creatinine, mg/dL	0.89 (0.21)	1.00 (0.22)	< 0.001	1.07 (0.30)	< 0.001
Serum cystatin C, mg/L	1.05 (0.28)	1.11 (0.30)	< 0.001	1.15 (0.29)	< 0.001

Data are presented as mean (SD).

Evaluable participants at baseline/Week 12/Week 24 were approximately 1217/519/682 for eGFR (CKD-EPI creatinine) and serum creatinine; 205/168/175 for eGFR (CKD-EPI cystatin C) and cystatin C; and 204/127/142 for eGFR (CKD-EPI creatinine–cystatin C). Paired baseline and Week 24 measurements were available in 135 participants for cystatin C-based eGFR and 108 for the combined creatinine–cystatin C equation.

CKD-EPI, Chronic Kidney Disease Epidemiology Collaboration; eGFR, estimated glomerular filtration rate.

a Paired comparison between baseline and Week 12.

b Paired comparison between baseline and Week 24 (paired *t*-test or Wilcoxon signed-rank test).

Applying the prespecified Week 24 renal criterion directly to the available safety-set data, 123 participants (10.0%) had either a serum creatinine increase ≥1.5 times baseline or an eGFR decrease ≥25%. Only 6 participants (4.9% of those eligible) completed the Week 36 off-treatment assessment. In these 6 participants, mean creatinine-based eGFR was 89.9 mL/min/1.73 m^2^ at baseline, 61.9 at Week 24, and 86.2 at Week 36; serum creatinine was 0.93, 1.29, and 0.98 mg/dL, respectively. Given the very small follow-up sample, these observations are descriptive only.

## 4 Discussion

In this multicenter real-world study, adding choline fenofibrate to statin therapy produced a substantial reduction in TG and remnant cholesterol and an increase in HDL-C. The modest LDL-C increase was accompanied by a lower sdLDL fraction and larger LDL particle size in the paired Lipoprint subset, while apoB did not change significantly in the smaller subset with paired measurements. These findings are consistent with lipoprotein remodeling, but the apoB and Lipoprint results should be regarded as supportive subset evidence rather than population-wide findings. Fenofibrate-mediated enhancement of triglyceride-rich lipoprotein catabolism can promote conversion of VLDL and remnant particles toward LDL, and larger, cholesterol-richer LDL particles may increase measured LDL-C without necessarily increasing particle number (Supplementary Fig. 6).

However, the cardiovascular outcome studies for fibrates in the statin era have generally yielded neutral or inconsistent results. Neither FIELD nor ACCORD-Lipid reduced the primary composite outcome in their overall populations [8], [9], and PROMINENT, the most recent large outcome trial with a selective PPAR-α modulator (pemafibrate), lowered TG substantially but did not improve cardiovascular outcomes in statin-treated patients with type 2 diabetes and hypertriglyceridemia [10]. Omega-3 fatty acid trials have been similarly discordant, with REDUCE-IT showing benefit from high-dose icosapent ethyl and STRENGTH showing no benefit with a mixed EPA/DHA preparation [26], [27]. One possible explanation is that TG concentration is a marker rather than the main driver of risk, and that the more relevant therapeutic target is the pool of apoB-containing particles rather than TG mass [28], [29], [30]. Our findings are compatible with this view and may also help explain why post-hoc analyses of fibrate trials have suggested greater benefit in patients with combined hypertriglyceridemia and low HDL-C, a group likely to have a greater burden of remnant lipoproteins and sdLDL [8], [9].

Our renal observations add to the literature on fenofibrate-associated changes in kidney function. Creatinine-based eGFR declined during treatment in the larger safety population. Cystatin C-based and combined estimates, available in much smaller subsets, changed in the same direction; this concordance is supportive but should not be overinterpreted given the limited availability of cystatin C. The magnitude of creatinine-based eGFR decline was similar in participants with and without hypertension and diabetes mellitus, and the sensitivity analysis restricted to participants without changes in background lipid-modifying therapy yielded a similar decline. These analyses reduce, but do not eliminate, concern that the renal signal was explained by these measured factors. Only 6 participants underwent off-treatment Week 36 assessment. Their renal indices moved toward baseline, but the sample is too small to support an inference regarding reversibility. Renal function should therefore continue to be monitored during fenofibrate treatment, particularly in patients with lower baseline eGFR, older age, or concurrent nephrotoxin exposure [31].

Our results should also be considered alongside newer TG-lowering therapies. ApoC-III- and ANGPTL3-targeted agents have shown larger reductions in TG and more consistent lowering of apoB and remnant cholesterol [32], [33], [34], [35]. Whether these differences translate into better cardiovascular outcomes beyond statin therapy is not yet known and is being tested in ongoing trials [29]. In current practice, fenofibrate still has a role in patients with persistent hypertriglyceridemia, and its effects are better interpreted through changes in apoB-containing lipoproteins than through LDL-C alone [28], [36].

This study has several limitations. First, the single-arm observational design without a concurrent comparator limits causal interpretation. Lifestyle counseling and clinically indicated treatment changes were not standardized by protocol. Although only 6.4% of participants with evaluable Week 24 medication records had a change in background lipid-modifying therapy and the sensitivity analysis restricted to those without such changes yielded results consistent with the primary analyses, changes in other treatments and lifestyle factors may still have contributed to the observed findings. Second, routine biochemical measurements were performed in local laboratories rather than a central reference laboratory; analytical platforms were not standardized and inter-laboratory variability was not measured. Third, the endpoints were surrogate lipid, metabolic, and renal markers rather than cardiovascular outcomes. Fourth, apoB and cystatin C were available only in subsets, introducing potential selection bias and limiting generalizability of conclusions based on these markers. Paired baseline and Week 24 measurements were available for 132 participants for apoB and 135 participants for cystatin C-based eGFR. The Lipoprint substudy was also availability-based and included paired measurements from 62 participants at 6 centers. Finally, only 6 participants completed the Week 36 off-treatment assessment, precluding any firm conclusion regarding reversibility. Cardiovascular-risk categories and lipid targets were defined according to Korean dyslipidemia guidelines and are not directly interchangeable with international primary-prevention risk-prediction systems such as SCORE2, PREVENT, or Framingham.

In conclusion, adding choline fenofibrate to statin therapy in patients with persistent hypertriglyceridemia produced marked reductions in TG and remnant cholesterol. In the paired Lipoprint subset, sdLDL fraction decreased and LDL particle size increased despite a modest rise in LDL-C; apoB did not change significantly in the smaller paired subset. Creatinine-based eGFR declined during treatment, with directionally similar findings in the smaller cystatin C subset. These findings support monitoring renal function and interpreting the lipid response beyond LDL-C alone.

## Credit author statement

Byung Jin Kim: Conceptualization; Data curation; Formal analysis; Funding acquisition; Investigation; Methodology; Project administration; Resources; Software; Supervision; Validation; Visualization; Roles/Writing - original draft; and Writing - review & editing. Hyeon Ju Jeong: Data curation; Formal analysis; Funding acquisition; Investigation; Methodology; Visualization; Supervision; and Writing - review & editing. Eun Seok Kang: Data curation; Funding acquisition; Investigation; Methodology; Project administration; Resources; Software; Supervision; Validation; and Writing - review & editing. Min-Woong Kim: Data curation; Funding acquisition; Investigation; Methodology; Visualization; Supervision; and Writing - review & editing. Sang-Hyun Kim: Data curation; Funding acquisition; Investigation; Methodology; Project administration; Resources; Software; Supervision; Validation; and Writing - review & editing. Yong-Hyun Kim: Conceptualization; Data curation; Funding acquisition; Investigation; Methodology; Project administration; Resources; and Writing - review & editing. Ki Hong Lee: Data curation; Funding acquisition; Investigation; Methodology; Project administration; Resources; Software; and Writing - review & editing.

Geu-Ru Hong: Conceptualization; Data curation; Funding acquisition; Investigation; Methodology; Project administration; Resources; Supervision; Validation; and Writing - review & editing. Young Sup Byun: Conceptualization; Data curation; Funding acquisition; Investigation; Methodology; Project administration; Supervision; Validation; and Writing - review & editing. Hoyoun Won: Data curation; Funding acquisition; Investigation; Methodology; Project administration; Resources; Software; and Writing - review & editing. Dong Heon Yang: Data curation; Funding acquisition; Investigation; Methodology; Project administration; Resources; Software; and Writing - review & editing. Jae Hyuk Lee: Data curation; Funding acquisition; Investigation; Methodology; Project administration; Resources; Software; and Writing - review & editing. Myung Soo Park: Data curation; Funding acquisition; Investigation; Methodology; Project administration; Resources; Software; and Writing - review & editing. Se Eun Park: Data curation; Funding acquisition; Investigation; Methodology; Project administration; Resources; Software; and Writing - review & editing. Sung Uk Kwon: Data curation; Funding acquisition; Investigation; Methodology; Project administration; Resources; Software; and Writing - review & editing. Si-Hyuck Kang: Data curation; Formal analysis; Funding acquisition; Investigation; Methodology; Project administration; Resources; Software; and Writing - review & editing. Min Seop Song: Data curation; Funding acquisition; Investigation; Methodology; Project administration; Resources; Software; and Writing - review & editing.

## Ethics declaration

Written informed consent to take part in the study and to publish the article has been obtained from all participants or their legal representatives. The privacy rights of participants have been observed.

This study included organ or tissue donors. This study includes human biological material and consent was obtained by donors, or their next of kin or legal representatives, for use in this study and for publication of the article. The samples used in this research were not sourced from executed prisoners or prisoners of conscience.

This study was performed in compliance with relevant laws, regulatory frameworks and guidelines where the research took place. This study was approved by the Kangbuk Samsung Hospital. (Approval No. IRB No. 2022-03-051)

## Data availability

The data analyzed in the present study are available from the corresponding author upon reasonable request.

## Declaration of generative AI use

During the preparation of this work, the authors used AI-assisted tools (Claude Sonnet 4.6, Anthropic) in order to improve language and readability. The authors independently performed the statistical analyses for this manuscript using the raw data, and all final tables and figures were prepared by the authors. The AI tool did not perform data analysis, make clinical judgments, or generate scientific hypotheses. After using these tools, the authors reviewed and edited the content as needed and take full responsibility for the content of the publication.

## Funding

This study was financially supported by Daewon Pharmaceutical Company in Korea. The study design was led by the investigators. Statistical analyses for the study report were conducted by a sponsor-funded coordinating research organization (CRO). For the present manuscript, the authors independently analyzed the raw data and interpreted the results. The sponsor had no role in the writing of the manuscript or in the decision to submit the article for publication.

## Declaration of competing interest

The authors declare the following financial interests/personal relationships which may be considered as potential competing interests:

Byung Jin Kim reports financial support was provided by Daewon Pharmaceutical Company. Hyeon Ju Jeong reports financial support was provided by Daewon Pharmaceutical Company. Eun Seok Kang reports financial support was provided by Daewon Pharmaceutical Company. Min-Woong Kim reports financial support was provided by Daewon Pharmaceutical Company. Sang-Hyun Kim reports financial support was provided by Daewon Pharmaceutical Company. Yong-Hyun Kim reports financial support was provided by Daewon Pharmaceutical Company. Ki Hong Lee reports financial support was provided by Daewon Pharmaceutical Company. Geu-Ru Hong reports financial support was provided by Daewon Pharmaceutical Company. Young Sup Byun reports financial support was provided by Daewon Pharmaceutical Company. Hoyoun Won reports financial support was provided by Daewon Pharmaceutical Company. Dong Heon Yang reports financial support was provided by Daewon Pharmaceutical Company. Jae Hyuk Lee reports financial support was provided by Daewon Pharmaceutical Company. Myung Soo Park reports financial support was provided by Daewon Pharmaceutical Company. Se Eun Park reports financial support was provided by Daewon Pharmaceutical Company. Sung Uk Kwon reports financial support was provided by Daewon Pharmaceutical Company. Si-Hyuck Kang reports financial support was provided by Daewon Pharmaceutical Company. Min Seop Song reports financial support was provided by Daewon Pharmaceutical Company. Reports a relationship with that includes:. Has patent pending to. If there are other authors, they declare that they have no known competing financial interests or personal relationships that could have appeared to influence the work reported in this paper.
