# Supplementary material for: Choline fenofibrate add-on to statins in persistent hypertriglyceridemia: efficacy, apoB-containing lipoprotein remodeling, and renal function
Source: Atheroscler Plus. 2026 Oct 30;66:100622. doi: 10.1016/j.athplu.2026.100622 (PMC13755848; doi:10.1016/j.athplu.2026.100622)
Supplement: Supplementary file 1 [file mmc1.docx]

**Supplementary Materials**

**Supplementary Table 1. Changes in lipid parameter ratios from baseline to Week 12 and 24**

| Variables | Baseline | Week 12 | P value* | Week 24 | P value** |
| --- | --- | --- | --- | --- | --- |
| TC/HDL-C | 3.60 [1.74–9.05] | 3.00 [1.49–8.77] | <0.001 | 3.00 [1.60–184.00] | <0.001 |
| LDL-C/HDL-C | 1.58 [0.02–6.30] | 1.54 [0.39–5.71] | 0.219 | 1.56 [0.25–152.60] | 0.389 |
| non-HDL-C/HDL-C | 2.60 [0.74–8.05] | 2.00 [0.49–7.77] | <0.001 | 2.00 [0.60–183.00] | <0.001 |
| TG/HDL-C | 6.37 [2.63–55.74] | 2.68 [0.51–83.21] | <0.001 | 2.64 [0.48–152.00] | <0.001 |
| ApoB/ApoA1 | 0.55 [0.26–1.02] | 0.63 [0.46–1.18] | 0.957 | 0.53 [0.25–1.38] | 0.980 |
| ApoB/LDL-C | 1.17 [0.34–2.46] | 0.97 [0.86–1.77] | 0.105 | 1.00 [0.70–2.75] | <0.001 |

Data are presented as median [range].

*P-value for the comparison between baseline and Week 12.

**P-value for the comparison between baseline and Week 24.

**Supplementary Table 2. Changes in metabolic profiles in the safety set**

| **Parameter** | **Baseline** | **Week 24** | **P value** |
| --- | --- | --- | --- |
| Fasting glucose, mg/dL | 126.2 (35.2) | 117.5 (36.2) | <0.001 |
| HbA1c, % | 6.76 (1.02) | 6.61 (0.95) | <0.001 |
| HOMA-IR | 3.53 (2.15, 5.52) | 2.80 (1.52, 4.37) | 0.015 |
| HOMA-β | 87.90 (50.51, 132.38) | 66.22 (41.69, 113.52) | 0.433 |
| QUICKI | 0.32 (0.30, 0.34) | 0.33 (0.31, 0.36) | 0.009 |

Data are presented as mean (SD) for FBS and HbA1c and as median (Q1, Q3) for HOMA-IR, HOMA-β, and QUICKI.
P values represent within-patient comparisons from baseline to Week 24 using the paired t-test for FBS and HbA1c and the Wilcoxon signed-rank test for HOMA-IR, HOMA-β, and QUICKI.
Only patients with both baseline and Week 24 values were included in the descriptive summaries and paired analyses. Paired sample sizes were n=514 for FBS, n=574 for HbA1c, and n=78 for HOMA-IR, HOMA-β, and QUICKI.
HbA1c, hemoglobin A1c; HOMA-IR, homeostasis model assessment of insulin resistance; HOMA-β, homeostasis model assessment of beta-cell function; QUICKI, quantitative insulin sensitivity check index.

**Supplementary Table 3. Adverse events in the safety set (n = 1,224)**

| **Event** | **Patients, n (%)** | **Events, n** |
| --- | --- | --- |
| Any adverse event | 90 (7.35) | 107 |
| Adverse drug reaction | 19 (1.55) | 20 |
| Serious adverse event | 9 (0.74) | 9 |
| Serious adverse drug reaction | 0 (0) | 0 |
| Adverse events of special interest |  |  |
| COVID-19 infection | 2 (0.16) | 2 |
| Ischemic stroke | 1 (0.08) | 1 |
| Target-vessel revascularization | 1 (0.08) | 1 |
| Most frequent individual events |  |  |
| Dizziness | 5 (0.41) | 5 |
| Glomerular filtration rate decreased* | 5 (0.41) | 5 |
| Hypoglycemia | 5 (0.41) | 6 |
| Dyspepsia | 4 (0.33) | 4 |
| Headache | 3 (0.25) | 3 |
| Diabetes mellitus inadequate control | 3 (0.25) | 3 |
| Chest pain | 3 (0.25) | 3 |

Coded using the MedDRA preferred term.

*Participants with a serum creatinine increase ≥ 1.5× baseline or an eGFR decrease ≥ 25% at Week 24 were eligible for an additional renal safety follow-up at Week 36.

**Supplementary Table 4. Changes in selected chemistry parameters**

| **Variables** | **Baseline** | **Week 12** | **P value** | **Week 24** | **P value** |
| --- | --- | --- | --- | --- | --- |
| ALT, U/L | 31.71 (16.72) | 27.90 (16.36) | <0.001 | 25.18 (15.65) | <0.001 |
| AST, U/L | 28.39 (10.98) | 29.16 (12.53) | 0.824 | 28.01 (14.01) | 0.019 |
| ALP, U/L | 73.06 (20.36) | 55.46 (17.84) | <0.001 | 53.68 (18.59) | <0.001 |
| Total bilirubin, mg/dL | 0.69 (0.31) | 0.64 (0.22) | 0.005 | 0.69 (1.61) | <0.001 |
| Uric acid, mg/dL | 5.64 (1.48) | 4.50 (1.79) | <0.001 | 4.49 (1.36) | <0.001 |
| CK, U/L | 117.57 (74.92) | 135.06 (97.28) | <0.001 | 132.22 (104.34) | 0.004 |
| LDH, U/L | 202.60 (74.21) | 190.27 (61.44) | 0.341 | 199.02 (74.83) | <0.001 |
| Fasting insulin, mU/L | 22.09 (28.86) | 43.09 (58.16) | 0.109 | 14.34 (19.22) | 0.007 |
| TSH, μIU/mL | 2.77 (5.29) | 6.96 (16.52) | 0.042 | 2.91 (3.28) | 0.884 |

Selected chemistry parameters were chosen on the basis of clinical relevance to safety; renal profiles are presented separately in Table 3.

Chemistry data are presented as mean (SD).

P values represent within-patient comparisons versus baseline using the paired t-test or Wilcoxon signed-rank test, as appropriate.

No imputation was applied for missing data and all analyses were based on observed data.

The number of evaluable participants at baseline / Week 12 / Week 24 was: ALT and AST, 1,205 / 559 / 725; ALP, 881 / 409 / 528; total bilirubin, 890 / 414 / 523; uric acid, 834 / 394 / 513; CK, 743 / 429 / 492; LDH, 257 / 112 / 131; fasting insulin, 171 / 15 / 138; and TSH, 198 / 35 / 72. The corresponding paired sample sizes for baseline-to-Week 12 / baseline-to-Week 24 comparisons were 557 / 718 for ALT and AST; 394 / 505 for ALP; 405 / 501 for total bilirubin; 381 / 480 for uric acid; 373 / 427 for CK; 98 / 98 for LDH; 7 / 86 for fasting insulin; and 22 / 35 for TSH.

ALP, alkaline phosphatase; ALT, alanine aminotransferase; AST, aspartate aminotransferase; CK, creatine kinase; LDH, lactate dehydrogenase; TSH, thyroid-stimulating hormone.

**Supplementary Table 5. Post hoc renal sensitivity analyses according to hypertension and diabetes mellitus status**

| Analysis | Group/covariate | n | Mean change or β (95% CI) | P value |
| --- | --- | --- | --- | --- |
| Stratified | Hypertension: No | 186 | −12.02 (10.25) | 0.930* |
| Stratified | Hypertension: Yes | 486 | −12.10 (9.96) |  |
| Stratified | Diabetes mellitus: No | 250 | −12.20 (9.88) | 0.582* |
| Stratified | Diabetes mellitus: Yes | 397 | −11.76 (10.21) |  |
| Multivariable | Baseline eGFR | 638 | β= −0.022 (−0.076 to 0.032) | 0.419 |
| Multivariable | Hypertension: Yes | 638 | β= −0.04 (−1.83 to 1.75) | 0.965 |
| Multivariable | Diabetes mellitus: Yes | 638 | β= +0.29 (−1.32 to 1.90) | 0.723 |
| Multivariable | Age, per year | 638 | β= +0.021 (−0.063 to 0.104) | 0.626 |
| Multivariable | Female sex | 638 | β= +0.93 (−0.79 to 2.66) | 0.288 |

*For the stratified analyses, the P value compares mean eGFR change between participants with and without the specified condition using Welch's t-test. Hypertension was defined as baseline systolic blood pressure ≥140 mmHg, diastolic blood pressure ≥90 mmHg, or antihypertensive medication use. Diabetes mellitus was defined as fasting plasma glucose ≥126 mg/dL, HbA1c ≥6.5%, or antidiabetic medication use. Classification was based on available baseline data; participants whose status could not be determined were excluded from the corresponding analysis. Within each subgroup, the change from baseline to Week 24 was significant (all p<0.001). The multivariable linear model included baseline eGFR, age, sex, hypertension, and diabetes mellitus; coefficients represent associations with change in eGFR (mL/min/1.73 m²). eGFR, estimated glomerular filtration rate; CI, confidence interval.

**Supplementary Table 6. Changes in background lipid-modifying therapy and sensitivity analysis**

**A. Background lipid-modifying therapy changes before the Week 24 assessment**

| Category | n/N (%) |
| --- | --- |
| FAS | 882 |
| FAS with Week 24 visit | 847 |
| Week 24 participants with evaluable medication data | 841/847(99.3) |
| No recorded lipid-modifying therapy change | 787/841(93.6) |
| Any lipid-modifying therapy change | 54/841(6.4) |
| Statin and/or ezetimibe-related regimen change* | 51/841(6.1) |
| Change limited to other lipid-modifying agents† | 3/841(0.4) |

**B. Sensitivity analysis in participants without changes in background lipid-modifying therapy**

| **Parameter** | **Paired n** | **Baseline** | **Week 24** | **Change** | **P value** |
| --- | --- | --- | --- | --- | --- |
| **Triglycerides, mg/dL** | **712** | **311.3 (145.7)** | **147.4 (159.7)** | **−163.8 mg/dL** | <0.001 |
| Remnant cholesterol, mg/dL | 547 | 39.8 (18.5) | 21.3 (10.4) | −18.5 mg/dL | <0.001 |
| non-HDL-C, mg/dL | 674 | 113.7 (29.4) | 100.8 (28.8) | −12.9 mg/dL | <0.001 |
| HDL-C, mg/dL | 699 | 44.0 (10.1) | 50.6 (12.4) | +6.6 mg/dL | <0.001 |
| **LDL-C, mg/dL** | **697** | **70.1 (27.0)** | **77.8 (30.8)** | **+7.6 mg/dL** | <0.001 |
| **ApoB, mg/dL** | **120** | **76.8 (16.8)** | **77.6 (17.7)** | **+0.8 mg/dL** | **0.959** |
| Serum creatinine, mg/dL | 633 | 0.894 (0.223) | 1.062 (0.300) | +0.168 mg/dL | <0.001 |
| **eGFR-Cr, mL/min/1.73 m²** | **633** | **88.3 (17.9)** | **76.2 (20.1)** | **−12.1 mL/min/1.73 m²** | <0.001 |

Values are mean (SD). Mean absolute change is Week 24 minus baseline and is expressed in the same unit as the corresponding parameter. Analyses include only participants with both baseline and Week 24 values for the respective variable.

*Other statin/ezetimibe regimen modifications included switching between statin-containing regimens, addition or discontinuation of ezetimibe, or treatment interruption/discontinuation. Switches between different statins were not classified as dose increases or decreases solely on the basis of milligram dose.

†Changes limited to a non-statin/non-ezetimibe lipid-modifying agent while the statin/ezetimibe regimen remained unchanged.

Medication changes were assessed from baseline to the Week 24 laboratory assessment. A medication newly started on the Week 24 visit date was not considered to have affected that visit’s laboratory results unless the preceding regimen had already ended. Paired t-tests or Wilcoxon signed-rank tests were used as appropriate; no missing values were imputed.

**Supplementary Fig. 1.** Study protocol schema with baseline and Week 12, 24, and 36 assessments; Week 36 was performed only in eligible participants after drug discontinuation

TG, triglyceride

**Supplementary Fig. 2. Disposition of analysis sets**

**Supplementary Fig. 3. Representative polyacrylamide gel electrophoresis pattern of lipoprotein subfractions at baseline and after 6 months of treatment**

(A) Pre-treatment (B) post-treatment

The pre- and post-treatment profiles are from the same participant. Migration of lipoproteins is shown from VLDL to HDL according to increasing density. Bands C, B, and A represent midbands corresponding to intermediate-density/remnant lipoproteins, and bands 1–7 represent LDL subfractions. In this participant, triglycerides decreased from 264 to 95 mg/dL and LDL-C increased from 51 to 82 mg/dL, while the sdLDL fraction decreased from 4.7% to 1.1% and mean LDL particle size increased from 263.6 to 270.5 Å. The profiles are provided as an illustrative example of redistribution toward larger LDL particles and are not intended as quantitative evidence by themselves.

Supplementary Fig. 4. Risk-stratified non-HDL-C and LDL-C goal attainment at baseline, Week 12, and Week 24 (very-high-, high-, moderate-, and low-risk groups per the 2018 Korean dyslipidemia guideline). Percentages were calculated using visit-specific evaluable denominators; participants with missing lipid values at a given visit were excluded from that visit’s denominator and were not classified as treatment failures. Values above bars are percentage (n/N).

HDL-C, high density-lipoprotein-cholesterol; LDL-C, low-density lipoprotein-cholesterol

**Supplementary Fig. 5. Changes in metabolic syndrome from baseline to week 24**

Full analysis set with MetS assessment at both visits (n = 840). McNemar’s test, p<0.001

MetS resolved in 395 of 715 participants with baseline MetS (47.0%)

MetS, metabolic syndrome.

**Supplementary Fig. 6. Proposed mechanism of lipoprotein remodeling with fenofibrate in statin-treated hypertriglyceridemic patients, annotated with the observed changes in the apoB-containing lipoprotein profile at Week 24. The sdLDL fraction and LDL particle-size values shown are based on participants with paired baseline and Week 24 Lipoprint measurements (n=62).**

C, cholesterol; IDL, intermediate-density lipoprotein; LDL, low-density lipoprotein; sdLDL, small-dense LDL; TG, triglycerides; VLDL, very-low-density lipoprotein
